# Virucidal or Not Virucidal? That Is the Question—Predictability of Ionic Liquid’s Virucidal Potential in Biological Test Systems

**DOI:** 10.3390/ijms19030790

**Published:** 2018-03-09

**Authors:** Julia Sommer, Susanne Fister, Tobias Gundolf, Birgit Bromberger, Patrick-Julian Mester, Anna Kristina Witte, Roland Kalb, Peter Rossmanith

**Affiliations:** 1 Christian Doppler-Laboratory for Monitoring of Microbial Contaminants, Institute of Milk Hygiene, Milk Technology and Food Science, Department for Farm Animal and Public Veterinary Health, University of Veterinary Medicine, Veterinärplatz 1, 1210 Vienna, Austria; Julia.sommer@vetmeduni.ac.at (J.S.); Susanne.fister@vetmeduni.ac.at (S.F.); Tobias.gundolf@vetmeduni.ac.at (T.G.); Patrick-julian.mester@vetmeduni.ac.at (P.-J.M.); Anna.witte@vetmeduni.ac.at (A.K.W.); 2 Institute of Milk Hygiene, Milk Technology and Food Science, Department for Farm Animal and Public Veterinary Health, University of Veterinary Medicine, Veterinärplatz 1, 1210 Vienna, Austria; Birgit.bromberger@vetmeduni.ac.at; 3 Proionic Production of Ionic Substances GmbH, 8074 Grambach, Austria; roland.kalb@proionic.com

**Keywords:** ionic liquid, disinfectant, virus, P100, MS2, Phi6, SARs, virucide

## Abstract

For three decades now, ionic liquids (ILs), organic salts comprising only ions, have emerged as a new class of pharmaceuticals. Although recognition of the antimicrobial effects of ILs is growing rapidly, there is almost nothing known about their possible virucidal activities. This probably reflects the paucity of understanding virus inactivation. In this study, we performed a systematic analysis to determine the effect of specific structural motifs of ILs on three different biological test systems (viruses, bacteria and enzymes). Overall, the effects of 27 different ILs on two non-enveloped and one enveloped virus (P100, MS2 and Phi6), two Gram negative and one Gram positive bacteria (*E. coli*, *P. syringae* and *L. monocytogenes*) and one enzyme (Taq DNA polymerase) were investigated. Results show that while some ILs were virucidal, no clear structure activity relationships (SARs) could be identified for the non-enveloped viruses P100 and MS2. However, for the first time, a correlation has been demonstrated between the effects of ILs on enveloped viruses, bacteria and enzyme inhibition. These identified SARs serve as a sound starting point for further studies.

## 1. Introduction

Ionic liquids (ILs), organic salts composed entirely of ions, have been successfully established over the past few decades as alternatives to organic solvents for various applications [1,2]. On account of their negligible vapour pressures, they were for a long time labelled “green solvents” and, thereby, environmentally preferable alternatives to conventional volatile organic solvents [3,4,5]. 

However, the “green solvent” attribute for ILs is now obsolete due to the growing database of (eco)toxicity data obtained from various biological systems, such as algae, fungi, plants, mammalian cells and bacteria [6,7,8,9,10,11,12]. Although it cannot be generalized that all ILs are hazardous, as there are so many combinations possible, an increasing number of studies investigated ILs as a new class of antimicrobials against a wide range of bacteria and fungi [11,13,14]. The latter findings are relevant to medical environments and the food industry [10,15,16,17,18]. As a consequence, third generation ILs as active pharmaceutical ingredients (APIs) have been recently developed [14,19,20]. The ready tunability of ILs permits these API-ILs to be designed specifically to match the physical and chemical properties of commonly used drugs (which are often administered as salts). These properties include stability, permeability, solubility and drug delivery [14,19,20,21,22]. Furthermore, some studies have shown that API-ILs are even effective against resistant bacterial strains and portend a solution to the current impasse in effective antimicrobials [20].

The limitations of today’s antibiotics and disinfectants, however, not only apply to resistant pathogenic bacteria and fungi, they also extend to viruses. However, not only do today’s antibiotics have limited or no efficacy against viruses, many commonly used disinfectants are useless, especially against non-enveloped viruses [23,24,25]. Some of the major public health threats today are infections by noroviruses (NoV) [26] and the hepatits B (HBV) and C (HBC) viruses [27]. However, in a rapidly changing world where even the food industry is becoming globalised, the threat of virus infections is increasing, as reports of virus-related outbreaks are confirmed [28,29]. These facts highlight the necessity to develop new virucidal disinfectants. 

Although ILs have already been utilized successfully for various antibacterial and antifungal applications, their virucidal potential has until now been neglected. To the best of our knowledge, our research group has been the only one thus far to investigate ILs specifically as possible virucides. In a previous study, we investigated the efficacy of various ILs against the non-enveloped viruses P100 and MS2, based on already known structure activity relationships (SARs) [30]. To our great surprise, none of the previously described IL toxicity SARs could be confirmed on these model viruses. ILs that had been reported to be highly active against a broad spectrum of bacterial and fungal pathogens turned out to be either completely inactive against both of these viruses or at least one of them. Nevertheless, results of this particular study are highly relevant as they demonstrate that existing SARs cannot be readily transferred to viruses. This is a serious setback because it implies that existing information based on enzymatic and cellular tests cannot be utilized for future virucidal applications of ILs.

**Figure 1:**
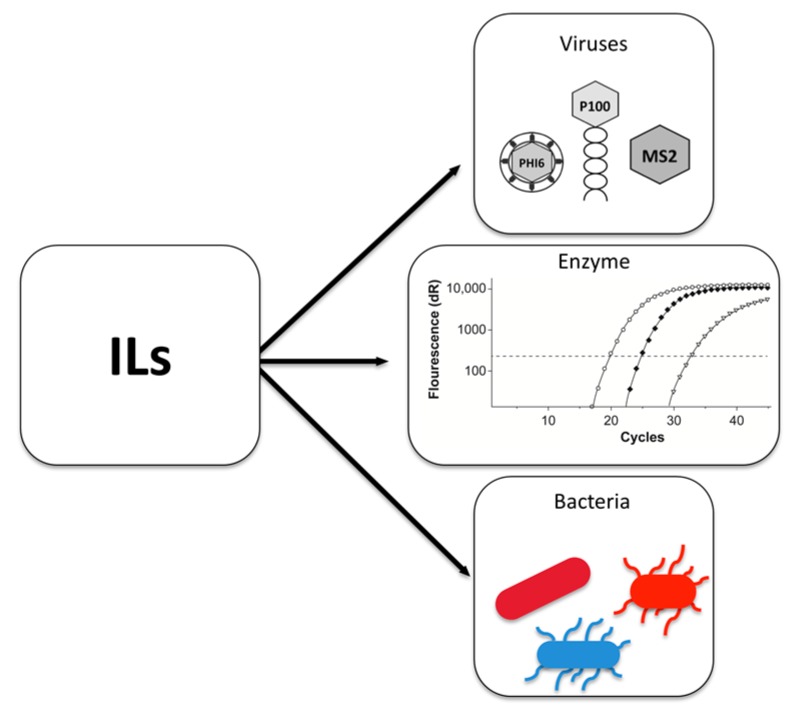
Overview of the biological test system. Virucidal effects of ionic liquids (ILs) (tested on viruses P100, MS2 and Phi6) and their toxicity (investigated by enzyme inhibition and minimum inhibitory concentration on the related host strains *L. monocytogenes*, *E. coli* and *P. syringae*) were compared and used to predict effects of ILs on viruses.

Before starting again from the ground up, we decided to conduct an extended systematic SAR analysis with a more holistic approach. In addition to the previously used non-enveloped viruses P100 and MS2, we included the virus Phi6. This *Pseudomonas syringae* (*P. syringae*) phage is an enveloped, double-stranded-RNA (ds-RNA) virus of the family *Cystoviridae* [31,32] that is commonly used as a surrogate for influenza viruses [33]. Supplementary to the virucidal tests, we set out to include antimicrobial testing of related host bacteria (*Listeria monocytogenes* (*L. monocytogenes*), *Escherichia coli* (*E. coli*) and *P. syringae*) as well as an enzyme inhibition test (based on quantitative real-time polymerase chain reaction (qPCR)) in order to investigate if and where the SARs diverge (Figure 1). Results could then be compared to provide information about the predictability of the virucidal impact of ILs.

## 2. Results and Discussion

Since IL cations and anions can be freely combined, a huge number of combinations are theoretically possible [34]. Clearly, structural components of both cations and anions are responsible for the physicochemical and toxic properties of ILs [35]. In this study, we performed a systematic analysis to determine the effects of specific structural arrangements of ILs on viruses. We determined the virucidal concentrations (VC) (using viruses P100, MS2 and Phi6), the minimum inhibitory concentration (MIC; using the corresponding bacterial host strains) and enzyme inhibition potential (using a qPCR assay) of 27 different ILs. Comparison of all data was insightful in respect of predicting the toxicity of ILs on viruses. 

### 2.1. Behaviour of Viruses to the Cationic Alkyl Side Chain Effect

Other published studies have already investigated the toxic effects of the structural elements of ILs on biological systems. Attention has been especially focused on the cation as the main component responsible for observed IL toxicity [36]. Particularly, the length of the alkyl side chains has been described as a major cause of toxicity [7,37,38,39,40,41]. This so-called “side chain effect”, referring to elongated side chains, has been correlated with higher lipophilicity [37,38,42].

**Figure 2:**
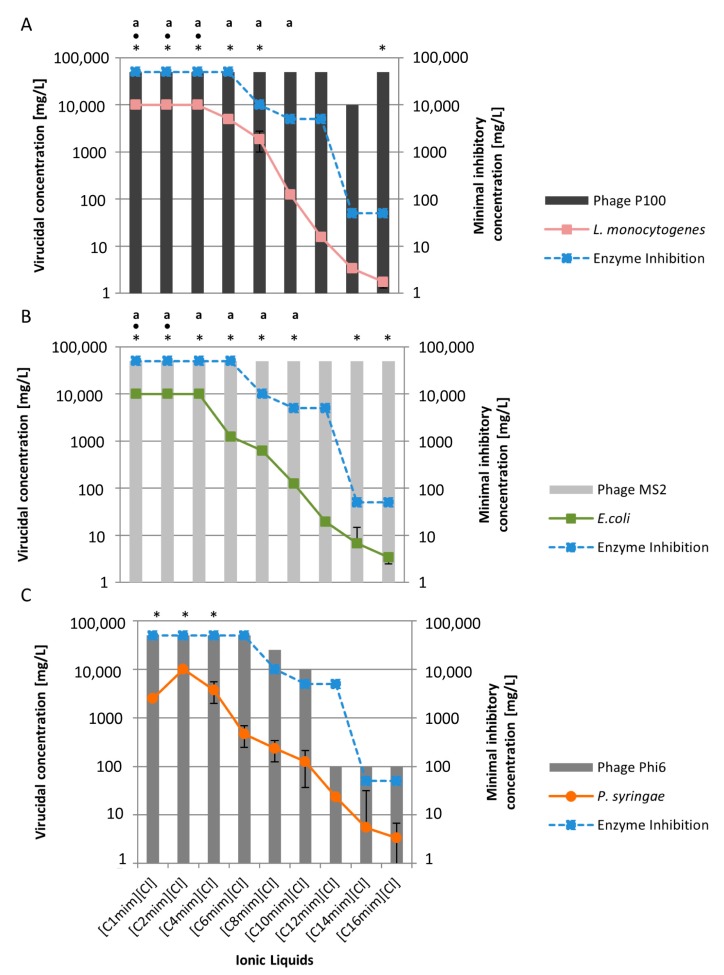
Virucidal concentration of imidazolium ionic liquids with increasing side chain length and the respective standard deviation using virus P100 (**A**); MS2 (**B**) and Phi6 (**C**) and the corresponding minimal inhibitory concentration of the host bacteria *L. monocytogenes* (**A**), *E. coli* (**B**) and *P. syringae* (**C**) and enzyme inhibition (blue line). (*) No virucidal effect was observed at a concentration of 50,000 mg/L. (•) No bactericidal effect was observed at a concentration of 10,000 mg/L. (^a^) calculation based on data of Fister et al. [30].

In this study, we investigated nine [C_n_mim][Cl]-based ILs with side chain lengths of C_1_–C_16_. We observed a clear side chain effect on the bacterial host strains *L. monocytogenes*, *E. coli* and *P. syringae* (Figure 2).

Side chain lengths of C_1_–C_4_ had no bactericidal effects but increasing side chain lengths to C_6_–C_16_ were associated with decreasing MICs (*L. monocytogenes* C_6_: 5000 mg/L–C_16_: 1.76 mg/L; *E. coli* C_6_: 1250 mg/L–C_16_: 3.39 mg/L; *P. syringae* C_6_: 468 mg/L–C_16_: 3.39 mg/L). Furthermore, the side chain effect was also seen in the enzyme inhibition test (C_8_: 10,000 mg/L–C_16_: 50 mg/L), demonstrating good accordance with bacterial and enzymatic toxicity data (Figure 2) [14,43,44].

However, with the viruses investigated, no such generalized conclusions can be drawn. For P100, ILs with a shorter side chain did not significantly reduce virus titres. Alternatively, when the length was increased to between C_10_–C_14_, the side chain length effect began to emerge (Figure 2A). The P100 outcome appears to indicate that a “cut-off “level, a defined side chain length where no toxicity increase can be observed [12,45], is reached with a side chain length of C_16_. This “cut-off” effect has already been observed in *Vibrio fischerii*, where no further increase in toxicity could be observed beyond a certain side chain length [12]. Another study reported a negative correlation between higher lipophilicity, due to an increase in side chain length, and decreased toxicity on different cell lines [46]. For the MS2 virus, IL side chain length had no virucidal effect within tested parameters (Figure 2B). Overall these results are in accordance with our previous study where we found similar trends for both viruses with IL side chain lengths up to C_10_ [30].

In contrast to these two non-enveloped viruses, the enveloped virus Phi6 showed higher sensitivity to ILs (Figure 2C). Short side chains (C_1_–C_4_) had no virucidal effect, but after starting from a side chain length of C_6_, recognizable virus reduction at 50,000 mg/L could be observed. With increasing side chain length, the required concentration of ILs to reach a virucidal effect decreased (C_8_: 25,000 mg/L; C_10_: 10,000 mg/L). ILs with side chain lengths between C_12_–C_16_ exhibited the strongest effect on virus Phi6 and only a concentration of 100 mg/L was necessary to decrease virus titre. These results demonstrate that the non-enveloped and enveloped viruses behave very differently. The most likely reason is the outermost layer of Phi6; a bilayer envelope consisting of protein and phospholipids.

Elongation of side chain length and thus higher IL lipophilicity is known to lead to increased toxicity and also conveys surfactant-like behaviour [39,47]. The latter results in non-specific breakdown of highly assembled lipophilic structures, such as biological membranes [47,48], which is presumably the reason for the sensitivity of enveloped viruses to lipophilic disinfectants [49]. 

In respect of the virucidal predictability of ILs, the most relevant observations were strong correlations between enzyme inhibition, antibacterial activity and virucidal activity against virus Phi6. This indicates a predictability of side chain length toxicity of ILs due to the enzyme inhibition test. In the case of the non-enveloped viruses P100 and MS2, no correlation between enzyme inhibition, antibacterial effects and virucidal activity were found.

### 2.2. Virucidal Activity Correlates with the Number of Cationic Side Chains

Similar to the effect of IL cationic side chain elongation, increasing the number of alkyl side chains also leads to enhanced lipophilicity [14,24]. The bactericidal effect of ILs with increasing numbers of side chains was also observed in 12 bacterial species [11]. Additionally, Byrne et al. [24] investigated the effects of ammonium-based ILs containing various numbers of side chains using the tobacco mosaic virus. They observed destabilization of the virus in the presence of ILs with three long alkyl side chains. In our previous study, varying the number of cationic alkyl side chains in the IL resulted in a diverse patterns of virus reductions [30].

The used ILs, [TMC_8_A][Cl], [DODMA][Cl] and [TOMA][Cl] (further defined in Table S3), have an ammonium-based cationic head and side chain numbers from one C_8_ chain to three C_8_ chains. In addition to the ammonium-based ILs, [C_10_C_10_im][Cl] (two long alkyl side chains) was tested (Table 1). The antibacterial and enzyme inhibition data are in good accordance with other published studies showing a correlation between the number of side chains and IL toxicity [11,50]. For both test systems, increasing the number of side chains decreased respective MICs.

Again, results with the non-enveloped viruses P100 and MS2 differed. Increasing the number of side chains did not affect the MS2 virus. While P100 was slightly affected by [TMC_8_A][Cl], [DODMA][Cl] as well as [C_10_C_10_im][Cl], no increase in virucidal activity with number of side chains could be observed. Surprisingly, even [TOMA][Cl] (three side chains) exhibited no effect. A so-called “cut-off” effect was already observed in other organisms [12,41,45] and it is possible that a similar effect is also observed here.

In contrast to results with these two non-enveloped viruses, the enveloped virus Phi6 showed higher sensitivity to ILs with increasing numbers of side chains. The more side chains an IL has, the lower the concentration required to produce a virucidal effect ([DODMA][Cl]: 10,000 mg/L; [TOMA][Cl]: 10,000 mg/L). As there is no further decrease of virucidal concentration from [DODMA][Cl] to [TOMA][Cl] on the enveloped virus Phi6, this could be due to a “cut-off” effect, again.

In the case of [C_10_C_10_im][Cl], a concentration of merely 100 mg/L was required to result in a reduction of ≥4 log_10_ units. This result could be due to the longer side chains with the increasing number of C-atoms [51] and due to increasing lipophilicity [45]. This increased lipophilicity is strong correlated with an enhanced membrane accumulation potential, resulting in higher toxic properties [45]. This effect was already observed on ammonium-based ILs with two longer side chains on *Pseudomonas putida* (*P. putida*). Interestingly, the toxicity was rapidly reduced with elongation of side chains from C_6_ up to C_8_–C_10_ [45]. Although this effect was seen on different bacteria [12,45], the results of the current study did not confirm that trend. On one hand, thess differences can be explained by the different structures of the tested ILs. In the current study, the cation core and the anion were different and that may influence the overall toxicity of the IL. On the other hand, in the current study, other bacteria were investigated. It is known that even at the species level, bacteria have different susceptibilities against ILs [11,52]. 

All in all, similar to the “side chain effect”, the results of enzyme inhibition, the MIC data of the three related host strains and results with virus Phi6 correlate well, while those for the non-enveloped viruses P100 and MS2 do not. 

### 2.3. Virus Inactivation Is Independent of Cationic Head Group

The effect of the cationic side chain length and the number of side chains can be considered the primary modulator of IL toxicity. However, it has been previously shown that the cationic head group can change the IL overall toxicity, although the effect was strongly dependent upon the test organism used (different cell lines, aquatic organisms and bacteria) [40,51,53]. For example, increasing the cationic head group’s overall lipophilicity, the addition of aromatic structures, the addition of nitrogen groups or cholinium-based cationic head groups have been shown to increase IL toxicity [36,48]. In this study, eight ILs with various cationic head groups, but similar cationic side chain lengths (between one and four alkyl chain lengths) and anions (either [Oac] or [Br]), were tested. There was only a slight toxic impact on bacteria and the target enzyme when [TBMA][Oac], [TBMP][Oac] and [Cholinium][Oac] were applied (Table S1). Our results indicate that none of the cationic head groups tested had a virucidal impact, neither on non-enveloped viruses, nor on the tested enveloped virus (Table S1). Although different functional groups were known to increase the toxicity of the cation, no clear SAR could be confirmed in this study. As described above, a side chain length between C_1_ and C_4_ had no influence on the viruses. Accordingly, the results obtained with the viruses were not surprising. In the future, testing different ILs cationic head groups with longer side chains might reveal if the cationic head group has an influence on IL virucidal potential. 

### 2.4. Anion Chaotropicity Does Not Exhibit Virucidal Activity

In comparison to the cation, the effect of the IL anion has been less well investigated. It was considered to play a minor role in overall toxicity [8]. However, it has now been shown that the choice of anion can indeed be a major factor responsible for IL toxicity, especially for those with less toxic cations [14]. For example, an overall increase in IL toxicity dependent on anion chaotropicity, in accordance with the Hofmeister series, has been demonstrated at an enzymatic level as well as with short-term exposure to bacteria [17,54]. In our previous study, no SAR-based anion chaotropicity could be found [30]. Nevertheless, we speculated that the chaotropicity of the IL anion could be especially promising for inactivation of enveloped viruses. Due to their lipid envelope, comparable to the bacterial membrane, inactivation through denaturation could be possible [40,55,56]. Table S2 shows the results of the seven investigated anions. 

In an earlier publication, Mester, Wagner and Rossmanith [17] observed that chaotropicity of the anion is one of the major factors responsible for an IL’s antimicrobial behaviour. Although in our present study, the effect was less pronounced, the data are in accordance with the previous study [17]. The strongest bactericidal effect was induced by [C_4_mim][I]. This is hardly surprising, as iodine is well known to be a potent antibacterial agent itself [49,57]. As expected, there was also a clear effect of the IL’s anion chaotropicity on the tested enzyme activity. 

Similar to the outcome of Fister et al. [30], our present study indicates that a chaotropic effect of the tested ILs on non-enveloped and enveloped viruses could also be excluded under the selected test conditions. Therefore, it might be possible that at higher IL concentrations, a chaotropic effect might also be seen on viruses. Nonetheless, concentrations of IL higher than 50,000 mg/L as disinfectants cannot be recommended on environmental grounds [2]. Another aspect which may be considered for viruses is the combination of ILs with longer cationic side chains and hydrophobic anions as the hydrophobic property of the anion could also increase ILs toxicity in different biological test systems including bacteria, as described in the literature [58].

To sum up, we performed a systematic analysis to determine the effects of specific structural components of ILs on viruses. We determined virucidal concentrations directly against viruses, estimated minimum inhibitory concentrations and the enzyme inhibition potential of 27 different ILs in order to investigate whether and, if so, how the SARs are related.

While the results of the antibacterial and enzyme inhibition tests coincided with all investigated SARs, this was not the case for all the viruses tested. In the case of the Phi6 virus, all cation SARs could be reproduced, while there was no observed effect of IL anion chaotropicity. 

Although some ILs were observed to be virucidal against both non-enveloped viruses studied, no clear SARs were observed. A slight side chain length effect and an effect of an increased number of side chains on virus P100 were recorded. However, there was neither a side chain effect nor enhanced IL toxicity associated with side chain number with virus MS2. Results of our study suggest that the IL effects on non-enveloped viruses, P100 and MS2, might instead depend upon as yet unknown factors, such as differences in particle size, capsid structure and genome structure. Similar differences regarding disinfectant effectiveness have previously been observed for phages MS2 and c2 [59]. However, the mechanism of action is still unclear. 

## 3. Material & Methods

### 3.1. Ionic Liquids

**Figure 3:**
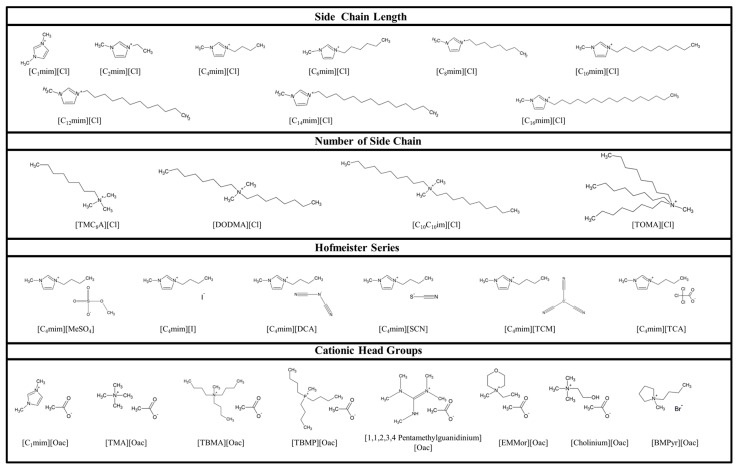
Structure of the 27 tested ILs, arranged according to their investigated structural characteristics.

Figure 3 shows the molecular structure of the 27 ILs used in this study (Figure 3 and Table S3). [C_2_–C_6_mim][Cl] and [C_4_mim][MeSO_4_, DCA, SCN and TCM] were provided by Merck KGaA (Darmstadt, Germany). [TMC_8_A][Cl], [DODMA][Cl], [C_1_mim][Cl], [C_4_mim][I and TCA] were synthesized using the CBILS^®^ (is a registered trademark of Proionic GmbH) route [60,61] as previously described by Fister et al. [62]. The precusor ILs used for synthesis of ([TMC_8_A] and [C_4_mim][MC]) were provided by Proionic GmbH (Grambach, Austria). Hydrochloric acid and trichloroacetic acid were obtained from Merck KGaA (Darmstadt, Germany), iodic acid was purchased from Sigma-Aldrich Chemie GmbH (Steinheim, Germany). [C_12_–C_16_mim][Cl] and [C_10_C_10_im][Cl] were purchased from Iolitec (Ionic Liquid Technologies, Heilbronn, Germany). All other ILs were provided by Proionic GmbH (Grambach, Austria). IL-solutions were prepared at concentration of 100,000 mg/L, 50,000 mg/L, 20,000 mg/L and 2000 mg/L, 200 mg/L, 20 mg/L and 2 mg/L (*w*/*v* in water). 

### 3.2. Phages and Host Strains

Virus P100, purchased as Listex™ P100 solution (Batch 12G26, Lot: 308; Micreos, Wageningen, The Netherlands), was used as a model for DNA viruses. Virus MS2, kindly provided by Regina Sommer, Medical University of Vienna, was used as a model for RNA viruses. Both viruses, P100 and MS2, were tested as surrogates for non-enveloped viruses, whereas virus MS2 was also used as a representative of human enteric viruses, such as human noroviruses [63]. Virus Phi6 was used to model enveloped viruses and as a surrogate for influenza viruses [33] and was purchased from the German Collection of Microorganisms and Cell Cultures (DSMZ, Braunschweig, Germany; DSM No. 21518). For enumeration and propagation of bacteriophage P100, MS2 and Phi6 their related strains were used. *L. monocytogenes* EGDe (ATCC BAA-679) was used for virus P100, *E. coli* Top 10F’ (Thermo Fisher Scientific, Waltham, MA, USA) was used for virus MS2 and *P. syringae* van Hall 1902 (DSMZ, DSM No. 21482) was used for virus Phi6. All bacteria strains were grown overnight in tryptone soya broth (TSB) with 0.6% (*w*/*v*) yeast extract (Oxoid Ltd., Hampshire, UK). *L. monocytogenes* EGDe and *E. coli* Top 10F’ were incubated at 37 °C overnight, whereas *P. syringae* was incubated at 25 °C overnight. Overnight cultures of *E. coli* Top 10F’ were diluted ten-fold in fresh medium and incubated at 37 °C for 3–4 h to gain a maximum number of viable cells in the logarithmic growth phase (log phase). For propagation of virus stocks (P100, MS2 and Phi6), the virus solutions were used for plaque assays (see Section 3.3) and plates with confluent lyses continued to be used. These plates were overlaid with 5 mL saline-magnesium (SM) buffer (5.8 g NaCl, 2.4 g Tris HCl, 1.0 g CaCl_2_, 0.1 g gelatine, add 1.0 mL H_2_O, pH 7.5) and shaken overnight at 4 °C. Afterwards the SM buffer was separated and centrifuged at 8000 rpm for 2 min. The supernatant was filtered (0.02 μm), aliquoted and stored at −20 °C.

### 3.3. Numeration of Plaque Forming Units

All viruses were used at concentrations of approximately 10^10^–10^11^ Plaque forming units (PFU)/mL. Virus titre of P100 and Phi6 was determined using the Small Drop Plaque Assay [64]. In short, first of all an overnight culture of either *L. monocytogenes* EGDe or *P. syringae* was 10-fold diluted in SM buffer [64,65]. Subsequently, a 10-fold serial dilution of the viruses was prepared in the bacteria-containing buffer. After 30 min of incubation at the 37° C (P100) or 25 °C (Phi6), 20 μL of each dilution were dropped on a tryptone soya agar (TSA)-plate and incubated overnight at 37 °C or 25 °C. Finally, plaques were counted and compared with the untreated controls. The Double Agar Overlay Assay was used for MS2 virus numeration [65]. In short, a 10-fold dilution series of the MS2 was prepared in SM buffer. Then an equal volume of log phase culture of *E. coli* Top 10F’ (OD_600_ nm 0.6–0.8) was added to each virus dilution. Afterwards 200 μL of this solution were mixed with 3 mL overlay medium (prewarmed TSB with 0.3% agar), and spread on a TSA-plate. When the overlay medium was hardened, the plates were incubated overnight at 37 °C. Finally, plaques were counted and compared with the untreated controls.

### 3.4. Determination of Virucidal Concentration (VC)

All viruses were mixed with each IL—solution to reach a final concentration of 1–50,000 mg/L (liquid ILs *v*/*v*; solid ILs *w*/*v* in water) and were incubated for 30 min at room temperature. Afterwards the viruses were numerated as described above (Section 3.3). The log_10_ reduction of virus concentration (plaque forming units (PFU)/mL), in comparison to the untreated control, was calculated. All ILs were initially tested at a concentration of 50,000 mg/L (*w*/*v* in water). ILs which caused a reduction that reached the detection limit of the assay or exhibited a reducing effect on virus titre concentrations of ≥4 log_10_ units were additionally tested at concentrations of 25,000, 10,000 and 1000 mg/L. ILs with particularly considerable impact on virus titre were further tested at 100, 10 and 1 mg/L. The VC was defined as the lowest concentration of the respective IL where a virus titre decreased by greater than 4 log_10_ units or the detection limit was reached.

All experiments were performed in duplicate on at least two different days (Small Drop Plaque Assay) or with at least two different dilutions with the Double Agar Overlay Assay on at least three different days. Each experiment included positive and negative controls. The detection limit was at least 4.5 log_10_ reduction (depending upon the virus used and on the ILs and their influence on the bacteria). 

### 3.5. Minimum Inhibitory Concentration (MIC) Assessment

The bactericidal impact of ILs was investigated by determination of the minimum inhibitory concentration (MIC). MIC of the examined ILs were determined by the serial two-fold dilution microtiter plate method in TSB-Y medium, according to Morrissey et al. [66]. Overnight cultures of *L. monocytogenes* EGDe and *E. coli* Top 10F’ were diluted 1:10 in 9 mL fresh media and incubated at 37 °C for 3 h to ensure a maximum number of viable cells in the log phase. For *P. syringae* overnight culture was used. Afterwards each dilution well of the microtiter plates (Corning Life Sciences B.V., Amsterdam, The Netherlands) was inoculated with 5 × 10^5^ CFU/mL of the respective bacterial cells and subsequently measured at a wavelength of 610 nm in a TECAN infinite F200 microplate reader (TECAN Austria GmbH., Groeding, Austria) to determine potential IL interference. After the first measurement the microtiter plates were then incubated for 24 h at the respective temperatures and thereafter absorbance was measured at 610 nm a second time to determine bacterial growth. The MIC was defined as the lowest concentration of the respective IL where no bacterial growth could be observed after 24 h. Each experiment included positive (inoculated growth medium) and negative controls (pure medium) and the experiments were performed at least three times on different days.

### 3.6. qPCR Enzyme Inhibition Assay

The qPCR enzyme inhibition assay was used in this study to determine the toxicological potential of ILs at an enzymatic level. The qPCR assay used was modified for the *prfA* gene of *L. monocytogenes* [67], using the non-specific DNA-dye EvaGreen^®^ (Jena Bioscience GmbH, Jena, Germany). One qPCR reaction of 25 μL final volume contained 2.5 μL of 10 × reaction buffer (Biozym Scientific GmbH, Hessisch Oldendorf, Germany), 500 nM of each primer (*LIP1*: GAT ACA GAA ACA TCG GTT GGC and *LIP2*: GTG TAA TCT TGA TGC CAT CAG G (both Eurofins Genomics, Ebersberg, Germany), 1.25 μM of probe EvaGreen^®^ (Jena Bioscience, Jena, Germany), 200 μM of each dNTPs (dATP, dTTP, dGTP and dCTP), 1 U of Taq DNA Polymerase (Biozym Scientific GmbH, Hessisch Oldendorf, Germany), ~150 copies of the internal amplification control *L. monocytogenes* EGDe and 5 μL of the IL. qPCR was performed in an Mx3000p real-time PCR thermocycler (Stratagene, San Diego, CA, USA) with initial denaturation at 94 °C for 5 min and amplification over 45 cycles at 94 °C for 15 s and 64 °C for 1 min. A final extension was performed for 1 min at 95 °C, 30 s at 55 °C and 30 s 95 °C. The MIC which was defined as the lowest concentration of the respective IL where no amplification was detected. All ILs were tested at final concentrations of 50,000, 10000, 2000 and 400 mg/L (*w*/*v*) in water. For the more active ILs, concentrations of 50, 0.5 mg/L and at least with 0.005 mg/L were tested. All experiments were performed at least twice on different days.in duplicate or triplicate.

## 4. Conclusions

In conclusion, the results of our study demonstrate that while virucidal effects of ILs on non-enveloped viruses occurred, previously described SARs are not applicable and future research must focus on identifying their actual mode(s) of action. In contrast to non-enveloped viruses, previously established SARs from biological model systems can be used as a sound starting point for further investigations of ILs on enveloped viruses. However, in order to facilitate the search for highly virucidal ILs that are universal against all virus types, further research must focus on concrete mechanisms of action.

## Figures and Tables

**Table 1:** Effect of the number of side chains investigated on three bacteriophages, related bacterial host strains and enzyme inhibition.

Effect of Number of Side Chain							
	Virucidal Concentration mg/L			Minimum Inhibitory Concentration mg/L			
Ionic Liquid	P100	MS2	Phi6	*L. monocytogenes*	*E. coli*	*P. syringae*	Enzyme
	≥4 Log_10_ Units	≥4 Log_10_ Units	≥4 Log_10_ Units				
[TMC_8_A][Cl]	10,000	50,000	10,000	729.17	1458.33	1041.67	50,000
	(10,000; 10,000)	(50,000; 50,000)	(10,000; 10,000)	(321.5; 1250)	(1250; 2500)	(625; 1250)	(50,000; 50,000)
[DODMA][Cl]	10,000 ^a^	>50,000 ^a^	1000	31.25	62.50	62.50	5000
	(10,000; 10,000)	(>50,000; >50,000)	(1000; 1000)	(31.25; 31.25)	(625; 625)	(62.5; 62.5)	(5000; 5000)
[C_10_C_10_im][Cl]	10,000	>50,000	100	1.43	3.39	3.65	50
	(10,000; 10,000)	(>50,000; >50,000)	(100; 100)	(0.8; <4)	(0.8; <4)	(1.6; 7.8)	(50; 50)
[TOMA][Cl]	>50,000 ^a^	>50,000 ^a^	1000	2.34	8.98	9.38	2750
	(>50,000; >50,000)	(>50,000; 50,000)	(1000; 1000)	(<0.78; <4)	(6.3; 7.8)	(<0.78; 15.6)	(5000; 500)

^a^ Calculation based on Fister et al. [30]. Mean virucidal concentration (VC) values (mg/L) and the span of measured values (lower limit; upper limit) are shown for the virus data. Mean MIC values (mg/L) and the span of measured values (lower limit; upper limit) are shown for bacterial and enzyme data.

## Supplementary Materials

Supplementary materials can be found at http://www.mdpi.com/1422-0067/19/3/790/s1.
