# Supplementary material for: Virucidal or Not Virucidal? That Is the Question—Predictability of Ionic Liquid’s Virucidal Potential in Biological Test Systems
Source: Int J Mol Sci. 2018 Mar 9;19(3):790. doi: 10.3390/ijms19030790 (PMC5877651; doi:10.3390/ijms19030790)
Supplement: Supplementary file 1 [file ijms-19-00790-s001.pdf]

International Journal of
Molecular Sciences

MDPI

Supplementary Materials: Virucidal or Not Virucidal? That Is the Question—Predictability of Ionic Liquid's Virucidal Potential in Biological Test Systems

Table S1. Effects of varying the cationic head group on three bacteriophages, related bacterial host strains and enzyme inhibition.

Effect of cationic head groups							
Ionic Liquid	Virucidal Concentration mg/L			Minimum Inhibitory Concentration mg/L			
	P100 ≥ 4 Log_{10} Units	MS2 ≥ 4 Log_{10} Units	Phi 6 ≥ 4 Log_{10} Units	*L. monocytogenes*	*E. coli*	*P. syringae*	Enzyme
[DMIM][Oac]	>50000 [a]	>50000	>50000	>10000	>10000	10000	50000
	(>50000; >50000)	(>50000; >50000)	(>50000; >50000)	(>10000; >10000)	(>10000; >10000)	(10000; 10000)	(50000; 50000)
[TMA][Oac]	>50000 [a]	>50000	>50000	>10000	>10000	10000	50000
	(>50000; >50000)	(>50000; >50000)	(>50000; >50000)	(>10000; >10000)	(>10000; >10000)	(10000; 10000)	(50000; 50000)
[TBMA][Oac]	>50000 [a]	>50000	>50000	10000	5000	5000	50000
	(>50000; >50000)	(>50000; >50000)	(>50000; >50000)	(10000; 10000)	(5000; 5000)	(5000; 5000)	(50000; 50000)
[TBMP][Oac]	>50000 [a]	>50000	>50000	10000	5000	3750	50000
	(>50000; >50000)	(>50000; >50000)	(>50000; >50000)	(10000; 10000)	(5000; 5000)	(2500; 5000)	(50000; 50000)
[1,1,2,3,4, Pentamethylguanidinium] [Oac]	>50000 [a]	>50000	>50000	2500	>10000	10000	50000
	(>50000; >50000)	(>50000; >50000)	(>50000; >50000)	(2500; 2500)	(>10000; >10000)	(10000; 10000)	(50000; 50000)
[EMMor][Oac]	>50000 [a]	>50000	>50000	10000	>10000	10000	50000
	(>50000; >50000)	(>50000; >50000)	(>50000; >50000)	(10000; >10000)	(>10000; >10000)	(10000; 10000)	(50000; 50000)
[Cholinium][Oac]	>50000 [a]	>50000	>50000	>10000	>10000	10000	40000
	(>50000; >50000)	(>50000; >50000)	(>50000; >50000)	(>10000; >10000)	(>10000; >10000)	(10000; 10000)	(50000; 10000)
[BMPyr][Br]	>50000 [a]	>50000	>50000	>10000	10000	2500	50000
	(>50000; >50000)	(>50000; >50000)	(>50000; >50000)	(>10000; >10000)	(10000; 10000)	(2500; 2500)	(50000; 50000)

[a] Calculation based on Fister et al. [30]. Mean virucidal concentration (VC) values [mg/L] and the span of measured values (lower limit; upper limit) are shown for the virus data. Mean MIC values [mg/L] and the span of measured values (lower limit; upper limit) are shown for bacterial and enzyme data

Table S2. Effects of IL anion chaotropicity tested on three bacteriophages, related bacterial host strains and enzyme inhibition.

Effect of Anions of the Hofmeister series							
Ionic Liquid	Virucidal Concentration mg/L			Minimum Inhibitroy Concentration mg/L			
	P100	MS2	Phi 6	*L. monocytogenes*	*E. coli*	*P. syringae*	Enzyme
	≥ 4 Log_{10} Units	≥ 4 Log_{10} Units	≥ 4 Log_{10} Units				
[C_4mim][Me SO_4]	>50000[a]	>50000[a]	>50000	>10000	>10000	7500	50000
	(>50000; >50000)	(>50000; >50000)	(>50000; >50000)	(>10000; >10000)	(>10000; >10000)	(5000; 10000)	(50000; 50000)
[C_4mim][I]	>50000[a]	>50000[a]	>50000	5000	1875	1250	50000
	(>50000; >50000)	(>50000; >50000)	(>50000; >50000)	(5000; 5000)	(2500; 5000)	(1250; 1250)	(50000; 50000)
[C_4mim][DC A]	>50000[a]	>50000[a]	>50000	>10000	10000	3750	50000
	(>50000; >50000)	(>50000; >50000)	(>50000; >50000)	(>10000; >10000)	(10000; >10000)	(2500; 5000)	(50000; 50000)
[C_4mim][SC N]	>50000[a]	>50000[a]	>50000	>10000	10000	3750	36667
	(>50000; >50000)	(>50000; >50000)	(>50000; >50000)	(>10000; >10000)	(10000; 10000)	(2500; 5000)	(50000; 10000)
[C_4mim][TC M]	>50000[a]	>50000[a]	25000	10000	5000	2500	10000
	(>50000; >50000)	(>50000; >50000)	(25000; 25000)	(10000; 10000)	(5000; 5000)	(2500; 2500)	(10000; 10000)
[C_4mim][TC A]	>50000[a]	>50000[a]	>50000	>10000	>10000	>10000	10000
	(>50000; >50000)	(>50000; >50000)	(>50000; >50000)	(>10000; >10000)	(>10000; >10000)	(>10000; >10000)	(10000; 10000)

[a] Calculation based on Fister et al. [30]. Mean virucidal concentration (VC) values [mg/L] and the span of measured values (lower limit; upper limit) are shown for the virus data. Mean MIC values [mg/L] and the span of measured values (lower limit; upper limit) are shown for bacterial and enzyme data

Table S3. Abbreviations and names of all tested ILs.

Ionic Liquids		
**Abbreviation**	**Cation**	**Anion**
[C_1mim][Cl]	Dimethylimidazolium	Chloride
[C_2mim][Cl]	1-Ethyl-3-methylimidazolium	Chloride
[C_4mim][Cl]	1-Butyl-3-methylimidazolium	Chloride
[C_6mim][Cl]	1-Hexyl-3-methylimidazolium	Chloride
[C_8mim][Cl]	1-Octyl-3-methylimidazolium	Chloride
[C_{10}mim][Cl]	1-Decyl-3-methylimidazolium	Chloride
[C_{12}mim][Cl]	1-Dodecyl-3-methylimidazolium	Chloride
[C_{14}mim][Cl]	1-Methyl-3-tetradecylimidazolium	Chloride
[C_{16}mim][Cl]	1-Hexadecyl-3-methylimidazolium	Chloride
[TMC_8A][Cl]	Trimethyoctylammonium	Chloride

[DODMA][Cl]	Dioctyldimethylammonium	Chloride
[$C_{10}C_{10}$Im][Cl]	1,3-Didecyl-2-methylimidazolium	Chloride
[TOMA][Cl]	Trioctylmethylammonium	Chloride
[C_4mim][$MeSO_4$]	1-Butyl-3-methylimidazolium	Methylsulfate
[C_4mim][I]	1-Butyl-3-methylimidazolium	Iodate
[C_4mim][DCA]	1-Butyl-3-methylimidazolium	Dicyanamide
[C_4mim][SCN]	1-Butyl-3-methylimidazolium	Thiocyanate
[C_4mim][TCM]	1-Butyl-3-methylimidazolium	Tricyanomethadine
[C_4mim][TCA]	1-Butyl-3-methylimidazolium	Trichloracete
[DMIM][Oac]	1-Di-3-Methylimidazolium	Acetate
[TMA][Oac]	Tetramethylammonium	Acetate
[TBMA][Oac]	Tributylmethylammonium	Acetate
[TBMP][Oac]	Tributylmethylphosphonium	Acetate
[1,1,2,3,4, Pentamethylguanidinium][Oac]	1,1,2,3,4, Pentamethylguanidinium	Acetate
[EMMor][Oac]	Ethyl-Methylmorpholinium	Acetate
[Cholinium][Oac]	Cholinium	Acetate
[BMPyr][Br]	1-Butyl-1-Methypyrrolidinium	Bromide
